# Supplementary material for: Machine Learning Interatomic Potentials for Reactive Hydrogen Dynamics at Metal Surfaces Based on Iterative Refinement of Reaction Probabilities
Source: J Phys Chem C Nanomater Interfaces. 2023 Dec 4;127(50):24168–82. doi: 10.1021/acs.jpcc.3c06648 (PMC10749455; doi:10.1021/acs.jpcc.3c06648)
Supplement: Supplementary file 1 — jp3c06648_si_001.pdf [file jp3c06648_si_001.pdf]

Supplementary Information to "Machine Learning Interatomic Potentials for Reactive Hydrogen Dynamics at Metal Surfaces Based on Iterative Refinement of Reaction Probabilities"

Wojciech G. Stark,[1] Julia Westermayr†,[1] Oscar A. Douglas-Gallardo‡,[1] James Gardner,[1] Scott Habershon,[1] and Reinhard J. Maurer[1,2,*]

[1]*Department of Chemistry, University of Warwick, Gibbet Hill Road, Coventry CV4 7AL, United Kingdom*

[2]*Department of Physics, University of Warwick, Gibbet Hill Road, Coventry CV4 7AL, United Kingdom*

I. STATISTICAL ERRORS EVALUATION

To calculate the statistical error of the sticking probability evaluation, based on 10,000 trajectories, we chose the following approach. First, we established 4 main groups, each containing 10,000 trajectories, and divided them into smaller subgroups, containing 500, 1,000, 2,000, and 2,500 trajectories (e.g. the first subgroup contains 20 subgroups of 500 trajectories). Next, we calculated sticking probabilities with the subgroups and the variance between them. Then we randomly shuffle the initial 10,000 trajectories and repeat the procedure 50 times. That allows us to obtain the linear fit of the inverse variance for the 10,000 trajectories (Fig. S1 (left)). Finally, the final error is obtained, by calculating the standard deviation. In Fig. S1 (right), we plotted the final sticking probabilities obtained using both SchNet and PaiNN models for H_2 (ν=0, J=1) Cu(111) in 925 K with the error bars that include only statistical error, without the uncertainties included in the main manuscript. The obtained statistical errors are negligible, unlike the model uncertainties received from evaluating the predictions using the committee of 3 models, based on different train/test splits.

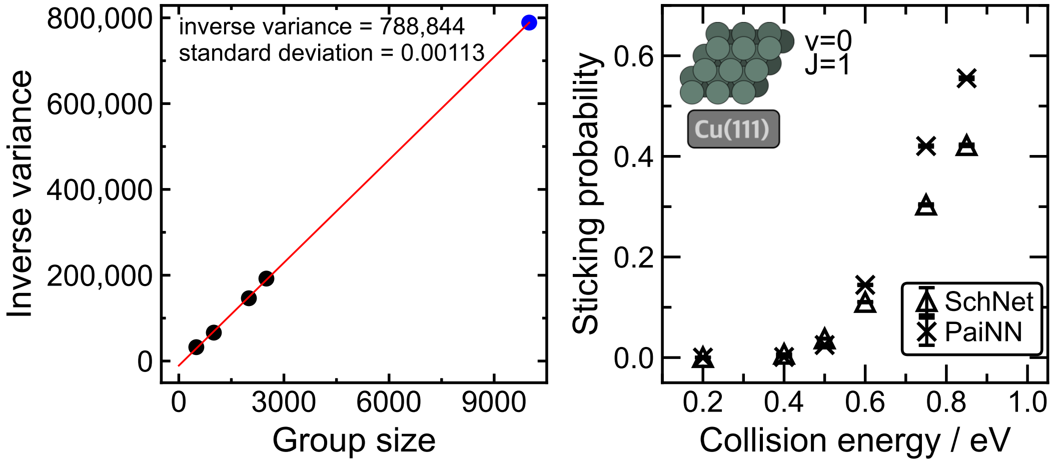

FIG. S1. **Statistical error evaluation.** The inverse variance of the final group size (10,000) predicted by linear regression fitted to four smaller group sizes (500, 1,000, 2,000, and 2,500) for collision energy of 0.6 (left side). Corresponding error bars for sticking probabilities predicted at different collision energies for H_2 (ν=0, J=1) dissociative adsorption on Cu(111) in 925 K (right side).

* r.maurer@warwick.ac.uk

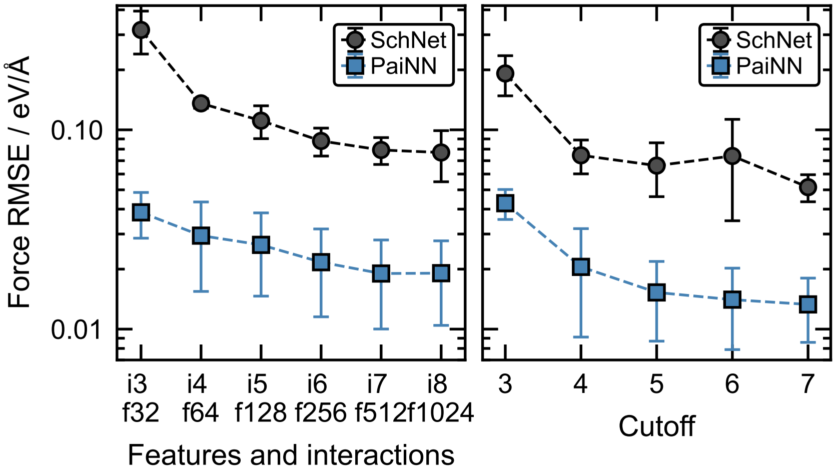

FIG. S2. **Optimization of model parameters.** The number of features and interaction blocks (left side) and the cutoff distance (right side) for non-equivariant (SchNet) and equivariant (PaiNN) MPNN models, based on the validation force RMSEs averaged over all of the cross-validation splits. The error bars represent the standard deviation between the force RMSEs obtained for all the splits. Optimization of the number of features and interaction blocks was done with the cutoff value of 4 Å and optimization of cutoff was done with 512 features and 7 interaction blocks.

II. MODEL PARAMETER OPTIMIZATION

We performed an optimization of different model parameters, such as the number of features, the number of interaction blocks, and the cutoff distance. The optimization was performed for both SchNet and PaiNN models independently.

On the left side of Fig. S2 the convergence of the number of features and interaction blocks for both models with respect to force RMSEs in log-scale is presented. SchNet converges slightly quicker towards the optimal number of features and interactions, however, the force errors are considerably higher than what is found with PaiNN. Both models converge with respect to the force RMSEs with around 6-7 interaction blocks and 256-512 features. In order to obtain reliable and accurate models, despite the slower evaluation times, we have chosen to use 7 interactions and 512 features in the final models for both codes.

Overall, the convergence of features and interaction blocks for both models is relatively slow. The models require a large number of features to capture many-body interactions well and consequently to provide accurate energies and forces. That may be due to the high average coordination of the atoms in the surface slab systems and the different surface terminations included in the database.

The right side of Fig. S2 shows a plot of the force RMSE in the logarithmic scale against the cutoff distance, revealing that although PaiNN achieves smoother and quicker convergence, a satisfactory level of convergence can already be achieved with a cutoff distance of 4 Å with both methods. The quick convergence towards a relatively low cutoff distance that allows modeling H_2 molecule dynamics on Cu surfaces indicates that the interaction between hydrogen and the surface atoms is well captured by the message passing through 7 layers and not much is to be gained by increasing the cutoff beyond the 4 Å range. Extending the cutoff further would also come with a significant increase in computational cost to evaluate the model.

III. DATABASE

The distribution of hydrogen atoms as a function of H-H and H-Cu distance is shown in Fig. S3. The figure shows that the distribution of H atoms in our database is very dense for the scattering simulations, all possible distances of hydrogen from the surfaces, as well as between the hydrogen atoms, are explored well.

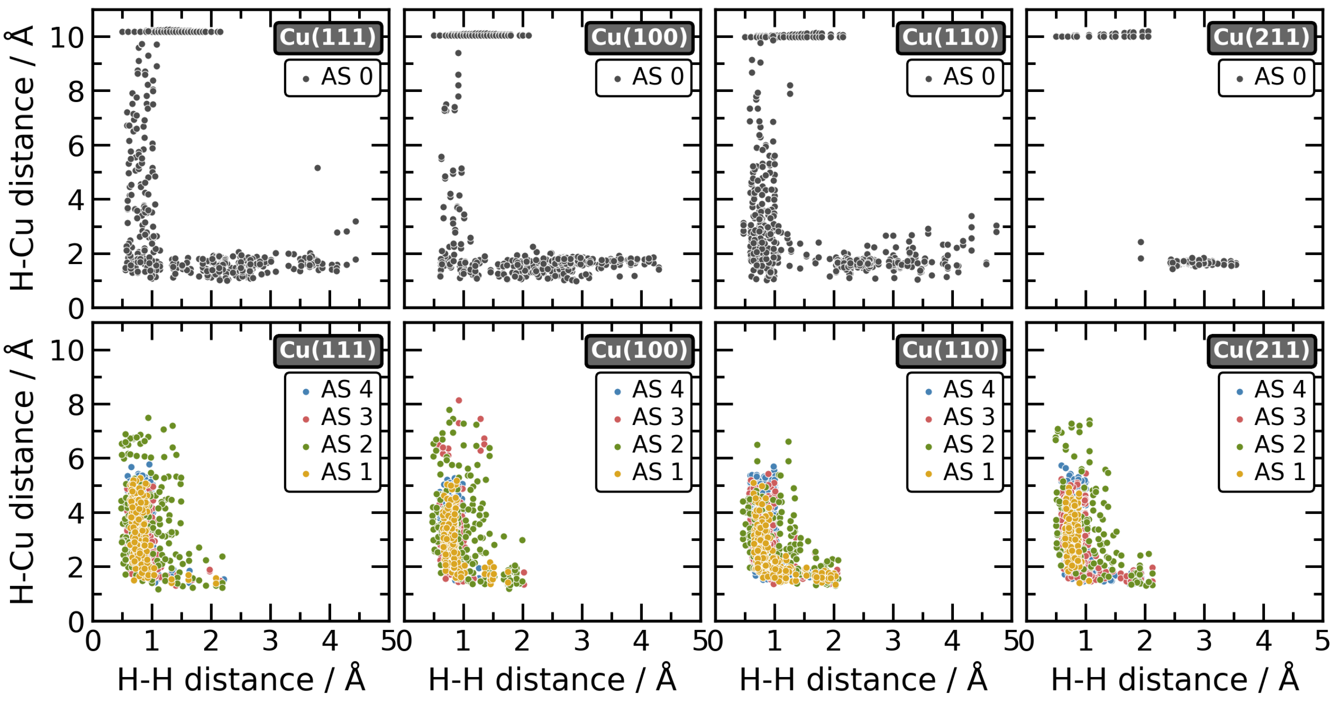

FIG. S3. **Distribution of hydrogen atoms depicted within H-H and H-Cu distances in our final database** The distribution includes datapoints from the initial database (*AS 0*) and following 4 iterations of adaptive sampling (*AS 1-4*). For calculations of H-Cu distance, we assume that Cu is the surface atom closest to the considered H atom. Note that the data points added through adaptive sampling (second row) are truncated at the H-H distance of 2.25 Å due to the stopping of dynamics trajectories.

IV. MINIMUM ENERGY PATHS

Minimum energy paths (MEP) were obtained using climbing image nudged elastic bands (CI-NEB) employing DFT, SchNet, and PaiNN (Fig. S4). The MEPs obtained with DFT match the MEPs obtained with PaiNN models very well, suggesting that barriers are modeled remarkably well by PaiNN models. However, the MEPs generated with SchNet confirm the findings shown in Fig. 7, where the PES generated with SchNet is not smooth. The instabilities in PES obtained with SchNet cause inaccurate MEP predictions (especially for Cu(110)). The higher number of images (50 instead of 20) used with PaiNN code, as opposed to SchNet code, was possible due to the much faster convergence of the NEB paths (fewer NEB steps needed to converge).

V. MODEL ERRORS

Fig. S5 shows energy and force predictions made with the final SchNet and PaiNN models compared to the reference DFT-based results. Energy RMSEs and MAEs correspond to the energy of an entire system, containing 56 atoms in H_2/Cu structures or 54 atoms in Cu surface-only structures.

VI. DEPENDENCE OF ENERGY WITH RESPECT TO THE COPPER LATTICE CONSTANT

The potential energy values obtained for specific lattice constant parameters calculated with DFT, SchNet, and PaiNN are shown in Fig. S6. We can clearly observe that energy predictions for different lattice constants are excellent. In contrast, the SchNet clearly struggles with the predictions, especially outside of the close range around the equilibrium lattice constant.

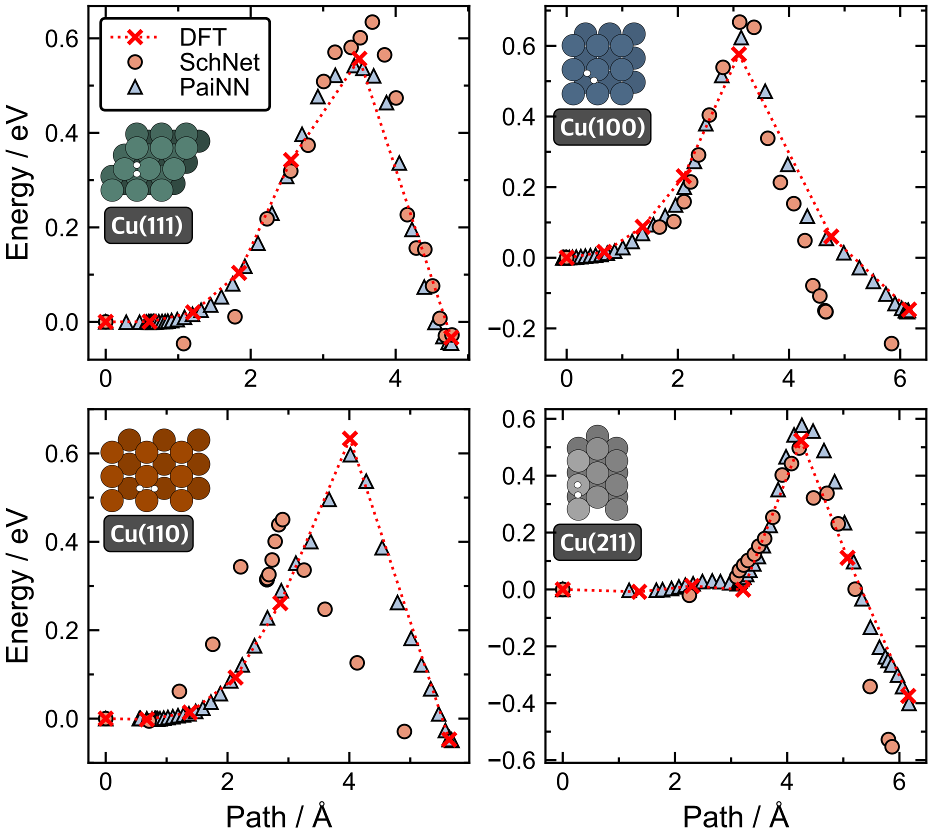

FIG. S4. **Minimum energy paths obtained using CI-NEB method for H_2 dissociative adsorption on different copper surfaces.** Potential energy is shown along the reaction path (Å), calculated using DFT (×) and both of the MLIPs included in our study: SchNet (orange circles) and PaiNN (grey-blue triangles). Figures that show top-down view of the systems at the transition states are included on the respective plots.

Present addresses:† **J.W.**: Wilhelm Ostwald Institute for Physical and Theoretical Chemistry, University of Leipzig, Leipzig 04103, Germany. ‡ **O.A.D.-G.**: Instituto de Ciencias Químicas, Facultad de Ciencias, Universidad Austral de Chile, Isla Teja, Valdivia 5090000, Chile.

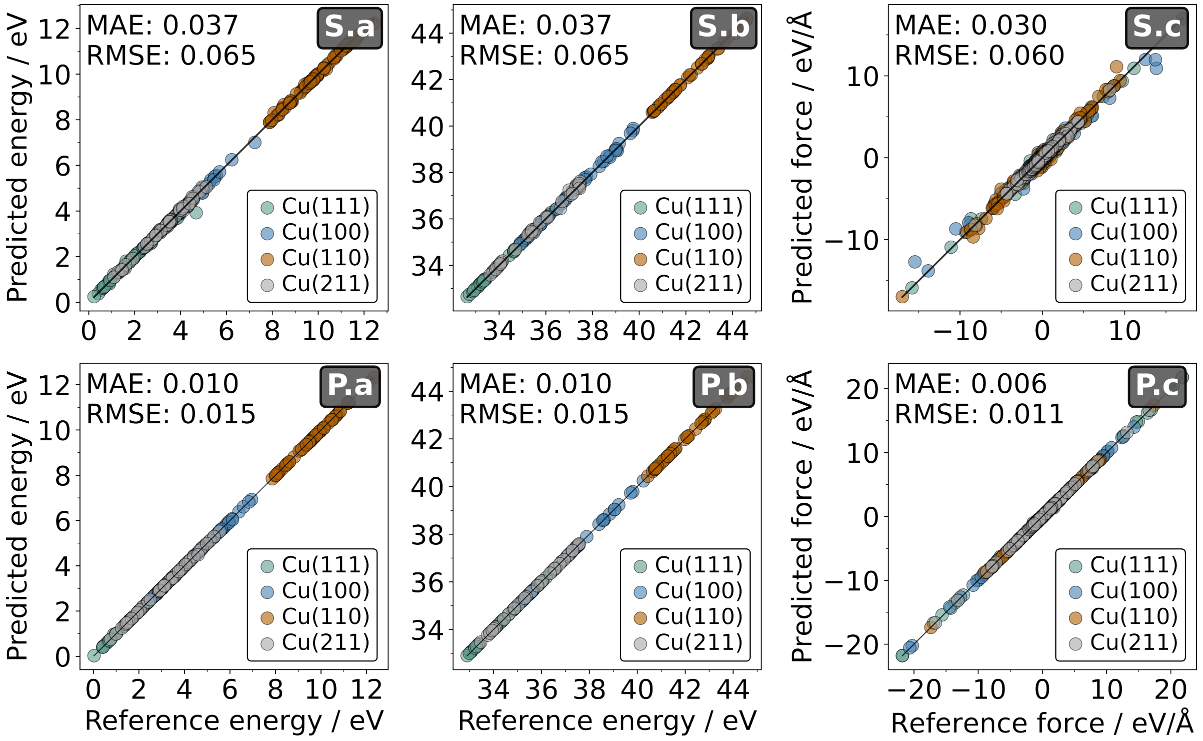

FIG. S5. **Energy (*a,b*) and force (*c*) predictions for H_2/Cu structures using SchNet (*S.a-c*) and PaiNN (*P.a-c*).** Best final models are compared to the reference (DFT) energies and forces. *S.a* and *P.a* show energies predicted for the structures with both H_2 and Cu atoms, *S.b* and *P.b* show energies predicted for just Cu surface structures. Force predictions include all of the structures together. The values of corresponding MAEs and RMSEs for energy (eV) or force (eV/Å) predictions are displayed in the upper left corner of every plot.

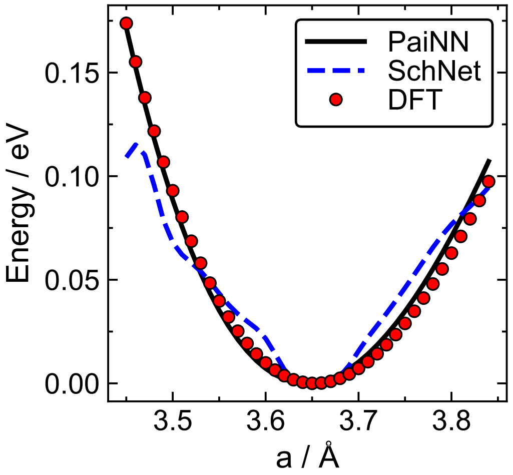

FIG. S6. **Relative potential energies of the Cu atoms for a set of lattice constants a (Å)**. Energies were calculated using a DFT code (red data points) and two MLIPs included in our study, namely, SchNet (blue, dashed line) and PaiNN (black, solid line).
